# Editorial: Modulation of immune function: drug discovery and translational application

**DOI:** 10.3389/fphar.2024.1495815

**Published:** 2024-10-16

**Authors:** Ruirong Tan, Junning Zhao

**Affiliations:** ^1^ Translational Chinese Medicine Key Laboratory of Sichuan, Sichuan-Chongqing Joint Key Laboratory of Innovation of New Drugs of Traditional Chinese Medicine, Sichuan Institute for Translational Chinese Medicine, Sichuan Academy of Chinese Medicine Sciences, Chengdu, China; ^2^ National Key Laboratory of Drug Regulatory Science, National Medical Products Administration (NMPA), Beijing, China

**Keywords:** drug, drug discovery, immune, immune regulation, immune response

In recent years, understanding how systemic immunity impacts disease development and therapeutic response has become one of the most compelling challenges in immunology and pharmacology (Hiam-Galvez et al., 2021; Spitzer et al., 2017). This Research Topic, “*Modulation of Immune Function: Drug Discovery and Translational Application*,” explores the intricate relationships between the immune system and various diseases, including cancer, neurodegenerative conditions, metabolic disorders, and infectious diseases. The articles in this Research Topic provide critical insights into the significance of immune modulation in disease occurrence and treatment, offering new perspectives on drug discovery and translational applications.

Immune dysregulation plays a central role in the development and progression of inflammatory diseases such as gouty arthritis. Modulating immune pathways can therefore be a key therapeutic strategy for controlling inflammation and reducing disease severity. Lang et al. present an example of traditional medicine’s therapeutic potential in immune modulation, focusing on Wuwei Shexiang Pill (WSP). Their research demonstrates that WSP not only ameliorates ankle swelling and inflammatory cell infiltration, but also modulates multiple immune pathways involved in inflammation. By employing an LC-MS-based metabolomics approach, the study identifies key metabolic pathways that WSP influences, offering a comprehensive view of how systemic immune modulation can alleviate inflammatory diseases. This also supports the idea that traditional medicines, when rigorously tested and characterized, can be valuable sources for drug discovery in immune-related conditions.

The modulation of immune responses is also crucial in addressing inflammatory diseases such as stroke (Macrez et al., 2011). Wang et al. delve into the neuroinflammatory mechanisms triggered by microglia following stroke, focusing on the therapeutic potential of Xiaoxuming Decoction Cutting Formula. Through comprehensive transcriptomic and metabolomic analyses, the study identifies miR-9-5p as a key mediator of post-stroke inflammation. This work provides a foundation for developing miRNA-based therapeutic interventions aimed at mitigating long-term neural damage after ischemic events. The potential to modulate neuroinflammation by targeting specific miRNAs represents an important step forward in developing precision medicine for neurological conditions.

Systemic immune responses are critical in shaping disease outcomes, particularly in conditions like cancer where immune evasion plays a central role in disease progression (Vinay et al., 2015). Yang et al. explore the traditional Chinese medicine Xihuang Pills (XHP) as a novel inhibitor of CYP3A4 in pancreatic cancer. CYP3A4 is an enzyme known for its role in drug metabolism, and its inhibition offers a new pathway for modulating the tumor microenvironment. XHP’s ability to interfere with the steroid hormone biosynthesis pathway underscores the therapeutic potential of integrating traditional medicines into modern pharmacology. Wang et al. contribute significantly to this Research Topic by exploring a novel deep learning segmentation model used for high-throughput drug screening in organoid-based systems. The model, called RDAU-Net, significantly enhances the accuracy of drug screening for bladder cancer by segmenting images of organoids more efficiently. This study highlights the increasing role of artificial intelligence and machine learning in pharmacological research, where computational tools can accelerate drug discovery processes by optimizing experimental models. Organoid systems, combined with AI-driven analysis, represent an exciting frontier in the development of targeted therapies, particularly in the context of personalized medicine.

Next-generation therapies including proteolysis-targeting chimeras (PROTACs), antibody-drug conjugates, and CAR-T cell therapy are revolutionizing the treatment to immune-related diseases (Li et al., 2022; Zhang et al., 2024; He et al., 2021). In this Research Topic, Wang et al. provide a comprehensive review of the latest advances in the use of antibody-drug conjugates (ADCs) for gynecologic cancers. ADCs have emerged as a promising class of targeted cancer therapies, particularly in cancers with low response rates to immune checkpoint inhibitors (ICIs). The article reviews ADCs like mirvetuximab soravtansine (IMGN853) and tisotumab vedotin, which are currently in clinical trials for ovarian and cervical cancers, respectively. By targeting specific tumor antigens and delivering cytotoxic agents directly to cancer cells, ADCs minimize damage to healthy tissues while enhancing the efficacy of treatment. This review underscores the evolving landscape of cancer immunotherapy and highlights the potential for ADCs to work in combination with other immunomodulatory treatments, such as ICIs, to overcome drug resistance and enhance patient outcomes.

In conclusion, the articles in this Research Topic offer a diverse and innovative exploration of how modulating immune function can lead to novel therapeutic strategies across a range of diseases. From the integration of traditional medicines with modern pharmacology to the use of artificial intelligence in drug screening, the contributions emphasize the importance of a multidisciplinary approach to drug discovery. These studies provide a roadmap for future research into immune modulation, focusing on the potential of multi-omics techniques, AI-driven methodologies, and the refinement of immunotherapy to tackle some of the most challenging diseases of our time. As the field of immune function modulation continues to expand, it is evident that both innovative and traditional approaches will play crucial roles in shaping the future of pharmacological interventions. The articles in this Research Topic exemplify the ongoing efforts to bridge the gap between basic immunology and translational medicine, driving forward the development of more precise, effective therapies for immune-related diseases.
